# Alpha-Enolase (ENO1) Correlates with Invasiveness of Cutaneous Melanoma—An In Vitro and a Clinical Study

**DOI:** 10.3390/diagnostics12020254

**Published:** 2022-01-20

**Authors:** Miriam Hippner, Michal Majkowski, Przemyslaw Biecek, Teresa Szkudlarek, Aleksandra Simiczyjew, Malgorzata Pieniazek, Dorota Nowak, Arkadiusz Miazek, Piotr Donizy

**Affiliations:** 1 Department of Clinical and Experimental Pathology, Division of Clinical Pathology, Wroclaw Medical University, 50-556 Wroclaw, Poland; miriam.hippner@gmail.com (M.H.); teresa.szkudlarek@umed.wroc.pl (T.S.); 2 Department of Biochemistry and Molecular Biology, Wroclaw University of Environmental and Life Sciences, 50-375 Wroclaw, Poland; arkadiusz.miazek@upwr.edu.pl; 3 Faculty of Biotechnology, University of Wroclaw, 50-383 Wroclaw, Poland; michal.majkowski@uwr.edu.pl; 4 Faculty of Mathematics and Information Science, Warsaw University of Technology, 00-662 Warsaw, Poland; przemyslaw.biecek@gmail.com; 5 Department of Cell Pathology, Faculty of Biotechnology, University of Wroclaw, 50-383 Wroclaw, Poland; aleksandra.simiczyjew@uwr.edu.pl (A.S.); dorota.nowak@uwr.edu.pl (D.N.); 6 Department of Oncology and Division of Surgical Oncology, Wroclaw Medical University, 53-413 Wroclaw, Poland; malgorzatapieniazek@interia.pl; 7 Department of Tumor Immunology, Hirszfeld Institute of Immunology and Experimental Therapy, Polish Academy of Sciences, 53-114 Wroclaw, Poland

**Keywords:** ENO1, cutaneous melanoma, melanoma cell lines, immunohistochemistry

## Abstract

**Simple Summary:**

Alpha-enolase (ENO1) undergoes accentuated overexpression in several solid cancers, but little is known about its status in cutaneous melanoma. The aim of this study was to investigate the prognostic significance of ENO1 in surgical resections from melanoma patients and to assess its expression and enzymatic activity in several melanoma cell lines. In clinical analysis, the overexpression of ENO1 in melanoma cells was significantly correlated with advanced clinical stage, presence of metastases in regional lymph nodes, and shorter cancer-specific overall survival and disease-free survival. We also demonstrated high expression of ENO1 in melanoma cell lines compared with normal melanocytes. Our study, which extends previous in vitro research, makes the alpha-enolase a candidate for a promising diagnostic and therapeutic target for various types of cancers. Consequently, additional testing of ENO1 as a target for melanoma therapy is necessary.

**Abstract:**

Alpha-enolase (ENO1) is a glycolytic metalloenzyme, and its overexpression occurs in numerous cancers, contributing to cancer cell survival, proliferation, and maintenance of the Warburg effect. Patients with an overexpression of ENO1 have a poor prognosis. The aim of the present study was to investigate the prognostic significance of ENO1 in surgical resections from 112 melanoma patients and to assess its expression and enzymatic activity in normoxia and hypoxia in several melanoma cell lines. Overexpression of ENO1 in tumor cells from patients was correlated with unfavorable prognosticators such as Breslow thickness, Clark level, mitotic activity, and the presence of ulceration. The expression of ENO1 also positively correlated with a greater thickness of the neoplastic infiltrate and a worse long-term prognosis for patients with cutaneous melanoma. We report significantly higher expression of ENO1 in melanoma cell lines in comparison to normal melanocytes. To conclude, our in vitro and clinical models showed that overexpression of ENO1 promotes invasiveness of melanoma cells and correlates with aggressive clinical behavior. These observations open the way to further search of a potential prognostic and therapeutic target in cutaneous melanoma.

## 1. Introduction

Cutaneous melanoma (CM) is an aggressive skin cancer whose incidence varies considerably between racial and ethnic groups of people. It is generally lower in people with highly pigmented skin chronically exposed to the sun. In Europe, this pattern is typical of the Mediterranean population, where the incidence approximates 5−7 cases/100,000 people [1]. In contrast, in Scandinavian countries and Switzerland, which have a prevalently fair-skin population and intermittent sun exposure, the incidence approximates 25−30 cases/100,000 people [2,3]. The mean CM incidence for the entire EU was 25 cases/100,000 people [4]. The major risk factor of CM, UV irradiation, not only depends on the geographical latitude, but also on the ozone layer depletion. A correlation is reported between a rise in CM incidence and a local thinning of ozone layer [5]. The mortality rate of CM is high, and it further raises with current environmental and lifestyle changes. Apart from cytostatic chemotherapies, new options for CM metastatic tumor treatment including BRAF (B-Raf protooncogene) and MEK (mitogen-activated protein kinase) inhibitors or their combination are available, but they often lead to appearance of chemoresistance [6]. Immunotherapy with anti-CTLA−4 (cytotoxic T-lymphocyte associated protein 4) or anti-PD−1 (programmed cell death 1) antibodies is generally superior to conventional chemotherapy, although its efficacy depends on the subtype of CM. For example, an anti-PD−1 therapy of uveal, acral, and mucosal melanoma had only limited efficacy with approximately 7%, 23%, and 32% of objective response rates, respectively [7,8]. Therefore, characterizing novel therapeutic targets involved in CM resistance to chemo- and immunotherapies may help to design new therapeutic strategies. One of such promising targets is alpha-enolase (ENO1, EC 4.2.1.11) [9]. It is an evolutionary conserved, glycolytic metalloenzyme responsible for the reversible dehydration of 2-phospho-D-glycerate to phosphoenolpyruvate. It functions as a homodimer, but may also assemble in supramolecular complexes with cytoskeletal, mitochondrial, or cell surface proteins displaying catalytic and “moonlighting” activities [10]. Despite being ubiquitous, ENO1 overexpression often reflects pathophysiological and metabolic status of the cell. An increase in ENO1 expression accompanies numerous human diseases (e.g., rheumatoid arthritis, systemic sclerosis, lupus erythematosus, Alzheimer’s disease), including over 18 classes of solid and hematological cancers [11,12,13,14,15].

Accumulated evidence demonstrates that, in the majority of cancers, ENO1 overexpression contributes to cancerous cell survival, proliferation, and the maintenance of the Warburg effect [9]. Mechanistically, both epigenetic regulators (e.g., DEAH-box helicase 33 (DHX33)-containing protein complex) and transcription factors (e.g., HIF−1α (hypoxia inducible factor 1 subunit alpha)) operate on an ENO1 promoter to increase ENO1 transcription during hypoxia—a predominant growth milieu of many cancers [16,17]. In ENO1-silenced tumor cell lines, the glycolysis rate diminishes in favor of the oxidative phosphorylation, but glucose influx remains high. This leads to the activation of the polyol-pathway consuming cellular NADPH and results in reactive oxygen species formation, which damage cell structures and contribute to the cancer cell senescence and death [18]. The available clinical data points to the poor prognosis and a worse overall survival of patients with increased ENO1 expression in glioma, pancreatic, lung, breast, colon, and bladder cancers [19]. However, in non-small cell lung cancers, ENO1 is downregulated at the protein level, whereas its expression on mRNA level remains elevated [20].

To our knowledge, comprehensive clinical assessment of ENO1 diagnostic and prognostic potential in cutaneous melanoma is not available in the literature. Previously, it was found that in five human skin melanoma cell lines (A375, MeWo, MEL-HO, Colo−800, and Colo−853), the RNA expression levels for ENO1 were upregulated 8−16 fold. Additionally, in the MeWo cell line treated with ascorbate, a reduction of ENO1 protein expression was documented [21]. The ascorbate-induced downregulation of ENO1 correlated with the reduced cell viability and in vitro migration capacity. 

The aim of the present study was to investigate the prognostic significance of ENO1 in surgical resections from 112 melanoma patients and to assess its expression and enzymatic activity in normoxia and hypoxia in several melanoma cell lines.

## 2. Materials and Methods

### 2.1. Cell Culture

Human epidermal melanocytes, adult (HEMa, 104−05A) and primary human epidermal melanocytes (lightly pigmented) (HEMn-LP, C0025C) were purchased from Cell Applications Inc (San Diego, CA, USA), and Cascade Biologics/Gibco (Carlsbad, CA, USA), respectively. Human melanoma cells lines Hs294T (HTB−140), A375 (CRL−1619), and WM9 (WM9−01−0001), WM1341D (WM1341D−01−0001) were acquired from the American Type Culture Collection (ATCC, Manassas, VA, USA), the European Collection of Authenticated Cell Cultures (ECACC, Porton Down, UK), and Rockland Immunochemicals, Inc. (Pottstown, PA, USA), respectively. A375 cells were cultured in DMEM (Hirszfeld Institute of Immunology and Experimental Therapy, Polish Academy of Sciences—HIIET, PAS, Wroclaw, Poland) containing 1 g/L glucose, 1.5 g/mL NaHCO_3_, 2 mM glutamine with 15% fetal bovine serum (FBS, Sigma–Aldrich, St. Louis, MO, USA). Hs294T, WM9 and WM1341D cells were cultured in DMEM (4.5 g/L glucose, 1.5 g/mL NaHCO_3_, 4 mM glutamine) (HIIET, PAS, Wroclaw, Poland) with 10% FBS (Sigma–Aldrich, St. Louis, MO, USA). Human epidermal melanocytes were grown in melanocyte growth medium (Cell Applications Inc, San Diego, CA, USA). All cells were cultured in 75 cm^2^ cell culture flasks (GoogLab Scientific, Rokocin, Poland) and were maintained at 37 °C in a humidified atmosphere containing 5% CO_2_. Cells were passaged twice a week using 0.25%/0.05% trypsin/EDTA solution (HIIET, PAS, Wroclaw, Poland). Cells were cultured either at normoxia (37 °C, 20.9% O_2_, and 5% CO_2_) or hypoxia (37 °C, 1% O_2_, and 5% CO_2_).

### 2.2. Cell Lysis

Human epidermal melanocytes and melanoma cells were trypsinized and washed twice with phosphate buffered saline (PBS). Next, the cells were resuspended in radioimmunoprecipitation lysis buffer—RIPA (50 mM Tris-HCl pH 7.4, 1% Triton X−100, 0.25% Sodium deoxycholate, 150 mM NaCl, 1 mM EDTA) supplemented with protease inhibitor cocktail (04693116001, Complete Protease Inhibitor Cocktail Tablets, EASYpack, Roche, Mannheim, Germany). After 30 min of incubation on ice, cell extracts were centrifuged at 16,000× *g* for 20 min at 4 °C. Supernatants were transferred into fresh tubes. The protein concentration in cell lysates was measured using BCA method (71285 Millipore, Burlington, MA, USA).

### 2.3. Western Blotting 

Supernatants containing 5 µg of total protein were denatured at 95 °C for 5 min with a Laemmli sample buffer containing 5% β-mercaptoethanol. Samples were separated using SDS-PAGE and transferred to the PVDF membranes (Millipore, Burlington, MA, USA). Next, the membranes were blocked with 5% skimmed milk in Tris-buffered saline with Tween 20 (TBST) overnight at 4 °C. Then, membranes were incubated for 1.5 h at room temperature (RT) with primary rabbit antibodies directed against ENO1 (PA5−13459 dilution 1:1000, Thermo Fisher, Waltham, MA, USA) and 1/2/3 Akt (sc−8312, H−136, dilution 1:500, Santa Cruz Biotechnology, Santa Cruz, CA, USA). Then, after three washes with TBST (Wash buffer), membranes were incubated for 1h at RT with the secondary anti-rabbit antibodies conjugated with horseradish peroxidase. Immunoblots were visualized using the G-Box gel doc system (Syngene, Frederick, MD, USA) and analyzed using ImageJ software (ver 1.53e, U. S. National Institutes of Health, Bethesda, MD, USA).

### 2.4. Immunofluorescence 

Melanoma cells were seeded on Millicell EZ slide (PEZGS0416, Millipore, Burlington, MA, USA) and after 12 h were fixed in 4% formaldehyde (FA) for 10 min at RT. Subsequently the cells were permeabilized with 0.1% Triton X−100 for 10 min at RT and then blocked with 2% BSA in PBS for 1h. Slides were incubated for 1h at RT with primary antibodies directed against ENO1 (dilution 1:50). The slides were then washed in PBS and incubated for 45 min with secondary anti-Goat IgG antibodies conjugated to fluorescein isothiocyanate (FITC) (554020, Becton Dickinson, Franklin Lakes, NJ, USA) and DAPI. The slides were washed in PBS and mounted with polyvinyl alcohol mounting medium with DABCO (10981 Sigma–Aldrich, St. Louis, MO, USA). 

A Stellaris 8 laser confocal scanning microscope equipped with 63x NA1.4 oil objective (Leica) was used to image samples. All images were taken at the same settings and further analyzed using FIJI software [22]. Images were filtered to remove noise (Median filter, radius = 2), then a triangle threshold was applied to segment cells from which mean fluorescence signals were measured. Data were exported to Excel software and analyzed with a *t*-test.

### 2.5. Enolase Activity Assay

Enolase activity was tested using the Enolase Activity Assay Kit (MAK178−1KT, Sigma-Aldrich, St. Louis, MO, USA). Cells were cultured on 6-well-plates, and were then homogenized according to the manufacturer’s instructions. Cell lysates were diluted and combined with a Reaction Mix. The plate was then incubated at 25 °C for 5−10 min, then the absorbance at 570 nm was measured using a Wallac Victor 2 1420 multi-label counter spectrophotometer (Perkin Elmer, Waltham, MA, USA) every 2−3 min, until the values approached the maximal value of standard curve. The calculation of the enzyme activity was conducted using the manufacturer’s instructions.

### 2.6. Immunohistochemistry

Tissue microarrays (TMAs) composed of three 1.5 mm tissue cores from each tumor were automatically constructed (TMA Grand Master, Sysmex, Warsaw, Poland). Immunohistochemical analysis was performed using rabbit polyclonal anti-ENO1 antibody (dilution 1:500) on 4-μm-thick paraffin sections mounted on silanized slides (Agilent DAKO, Santa Clara, CA, USA). The slides underwent automated dewaxing, rehydration, and heat-induced epitope retrieval with EnVision Target Retrieval Solution (Agilent DAKO, Santa Clara, CA, USA) for 30 min at 97 °C in PT Link Pre-Treatment Module for Tissue Specimens (DAKO). Liquid Permanent Red (Agilent DAKO, Santa Clara, CA, USA) was utilized as a detection system. Human breast and pancreatic adenocarcinomas were stained as positive controls. Negative controls were processed using FLEX Rabbit Negative Control, Ready-to-Use (Agilent DAKO, Santa Clara, CA, USA) in place of the primary antibody.

Scoring of ENO1 immunostains was performed using the H-score [(percentage at 1+) × 1 + (percentage at 2+) × 2 + (percentage at 3+) × 3], which integrates the intensity and percentage of positive cells into a combined score. The median H-score (200) was used as a cut-off value for high (H-score > 200) and low ENO1 (H-score ≤ 200) expression [23].

### 2.7. Patients

We analyzed 112 cutaneous melanoma patients treated at the Regional Oncology Centre in Opole, Poland, diagnosed between 2005 and 2010. Patients were enrolled in this study based on the availability of medical documentation and paraffin blocks with primary tumors. Comprehensive clinical data were retrieved from the archival medical records (Regional Oncology Centre, Opole, Poland).

This study was reviewed and approved by the ethics committee of the Wroclaw Medical University, Wroclaw, Poland (No. 277/2020). The patients did not personally participate in the study, and the results of these investigations did not have any influence on the original treatment of patients since it had already finished. All investigations were performed in accordance with the Declaration of Helsinki.

Clinical parameters included in this study were age, gender, location of the primary tumor, regional nodal status (including the information of sentinel lymph node biopsy (SLNB) procedures), presence or absence of distant metastases, and information concerning disease recurrence. Based on hematoxylin and eosin (H&E) staining from sections of archival formalin-fixed paraffin-embedded tumor specimens, we evaluated detailed histopathologic parameters: Breslow thickness, Clark level, histological type, mitotic rate (number of mitotic figures per 1 mm^2^), presence of ulceration, lymphangioinvasion, microsatellitosis, intensity of tumor-infiltrating lymphocytes (TILs), and microscopic evidence of regression. pT stadium was evaluated according to the pathologic stage classification (pTNM, AJCC 8th edition) as follows: pT1: melanoma 1.0 mm or less in thickness, pT2: melanoma >1.0 to 2.0 mm in thickness, pT3: melanoma >2.0 to 4.0 mm in thickness; and pT4: melanoma >4.0 mm in thickness.

### 2.8. Statistical Analysis

Statistical analysis of parameters from the clinical and histopathologic evaluation was performed using R language [R Core Team. R: A language and environment for statistical computing. R Foundation for Statistical Computing, Vienna, Austria, https://www.r-project.org/ (2019, accessed on 12 March 2021)] and the Survminer tool [24]. For the purposes of correlation analysis, we assumed a dichotomous division of ENO1 expression into low and high corresponding to a semiquantitative H-score of ≤200 and >200, respectively. Kaplan–Meier curves and the log-rank test were used to determine the cancer-specific overall survival (CSOS) and disease-free survival (DFS); all analyses were carried out using the survival package for R [R Core Team. R: A language and environment for statistical computing. R Foundation for Statistical Computing, Vienna, Austria, https://www.r-project.org/ (2019, accessed on 12 March 2021); [24]. The Wilcoxon two-sample test was used to determine the correlations between the ENO1 expression and continuous variables. ENO1 expression and binary variables were determined using Fisher’s exact test, while the correlations with other categorical variables were analyzed using the chi-square test.

## 3. Results

### 3.1. Expression of ENO1 in Melanoma Cell Lines

**Figure 1:**
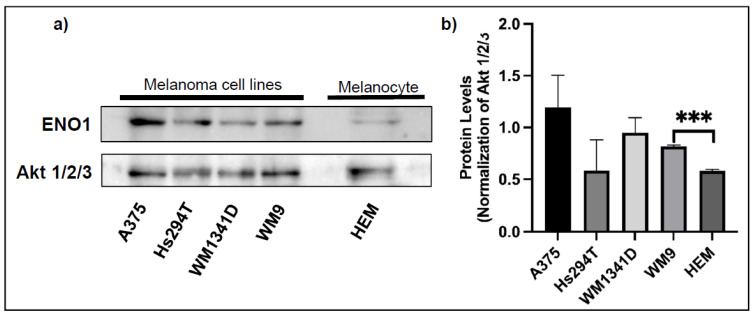
The expression level of ENO1 in the cell lysates from primary melanocytes and melanoma cell lines. Representative Western blots showing ENO1 and Akt 1/2/3 expression (for normalization) in protein lysates obtained from the primary human melanocytes (HEM) and indicated melanoma cell lines (**a**). Densitometric ENO1/Akt ratios are shown as mean values (*n* = 3 except for HEM, *n* = 2) ± standard error of the mean (SEM) (**b**). The significance level was set at *p* = 0.001–0.0001 (***).

**Figure 2:**
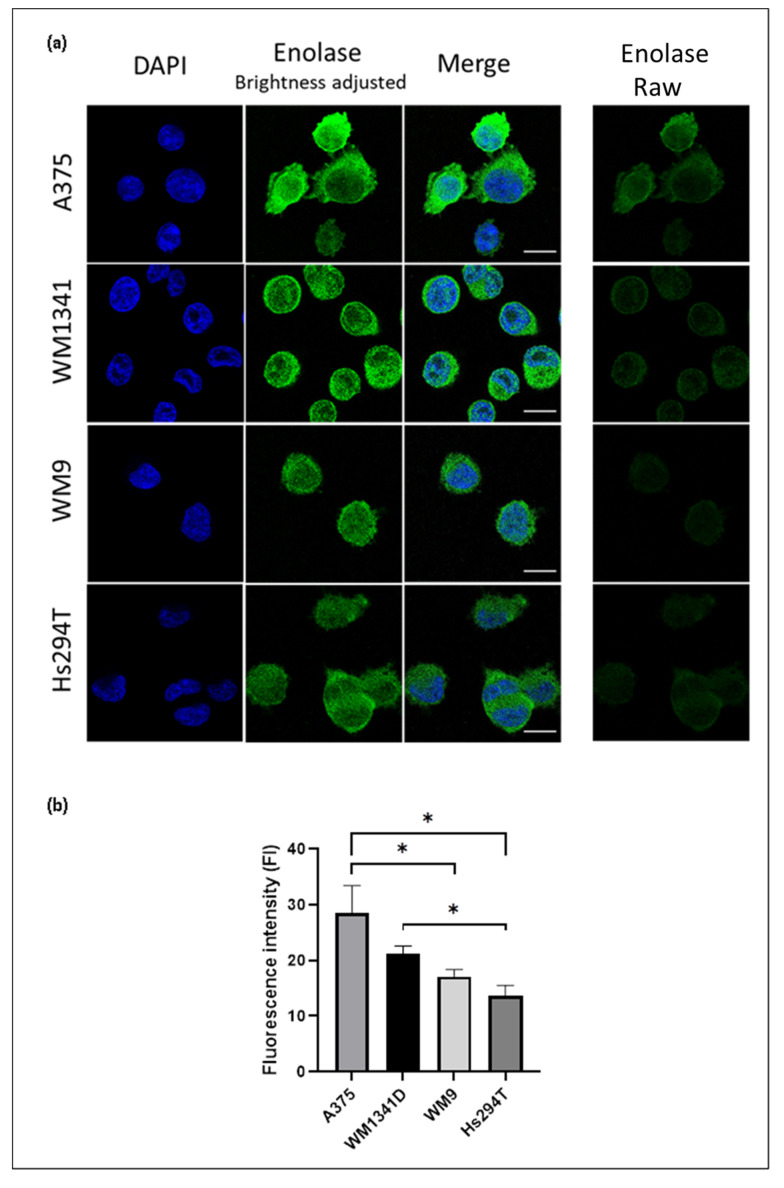
Enolase expression in melanoma cell lines determined by indirect immunofluorescence and confocal microscopy. (**a**) Single optical sections showing cells stained for ENO1 (green) and DAPI (blue). In the second column ENO1 signal was enhanced by brightness adjustment for the sake of better visualization. Raw images (shown in the last column) were subjected to fluorescence signal intensity analysis. Bar—15 μm. (**b**) Fluorescence signal intensity of the ENO1 presented as a mean ± standard deviation. The significance level was set at *p* = 0.05–0.01 (*). Subsequent number of cells were analyzed: A375—40 cells, WM1341D—71 cells, WM9—25 cells, Hs294T—45 cells.

We analyzed the expression of ENO1 in four cell lines—two derived from the primary tumor (A375 and WM1341D), and two from lymph node metastases (Hs294T and WM9). Western blotting and semi-quantitative immunofluorescence analyses corroborated similar pattern of ENO1 expression (Figure 1 and Figure 2, and Supplementary Figure S1 and Supplementary Table S1) with A375 and Hs294T cells being the highest and the lowest expressors, respectively. We observed a statistically significant (*p* = 0.001) upregulation of ENO1 in the WM9 cell line in comparison with the primary human epidermal melanocytes (Figure 1). Interestingly, this expression pattern of ENO1 also positively correlated with estimated population doubling times of examined melanoma cell lines (data not shown). Signal for ENO1, analyzed by immunofluorescence, was primarily localized in the cells’ cytoplasm (Figure 2). 

### 3.2. ENO1 Enzymatic Activity in Melanoma Cell Lines

**Figure 3:**
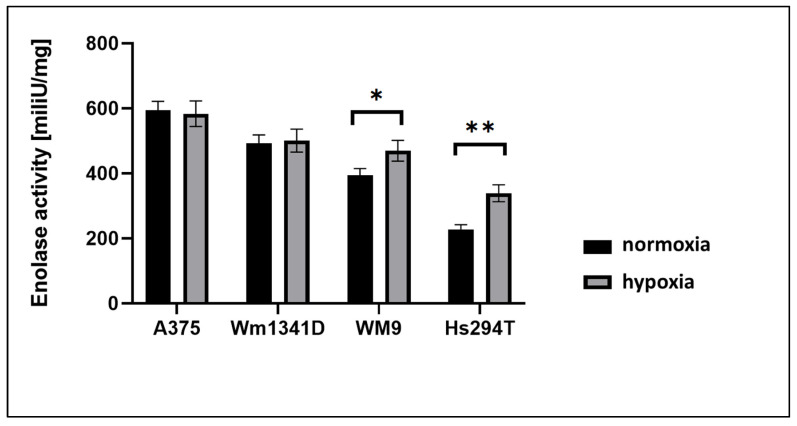
The enolase activity in protein lysates prepared from A375, Hs294T, WM1341D, and WM9 melanoma cells cultured under normoxia or hypoxia. Bars represent mean values (*n* = 4) ± standard error of the mean (SEM). The significance level was set at *p* = 0.05–0.01 (*), *p* = 0.01–0.001 (**).

To assess ENO1 activity, we performed enzymatic assay on protein lysates from melanoma cells. We used the H_2_O_2_ standard curve (Supplementary Figure S2) to calculate the results of enolase activity in the cell lines. The results, shown in Figure 3 and Supplementary Table S2, correlated with patterns of ENO1 expression (Figure 1 and Figure 2), suggesting that ENO1 enzymatic activity is proportional to ENO1 expression in melanoma cell lines used in this study. Moreover, when measured under hypoxia, the ENO1 activity was significantly increased in two cell lines derived from lymph node metastases (WM9, Hs294T), but not in lines from primary skin lesions (A375, WM1341). This result suggests that increased ENO1 activity under hypoxia, better reflecting lymph node milieu, may provide additional survival advantage to metastatic cells.

### 3.3. Expression of ENO1 in Cutaneous Melanoma Patients

**Figure 4:**
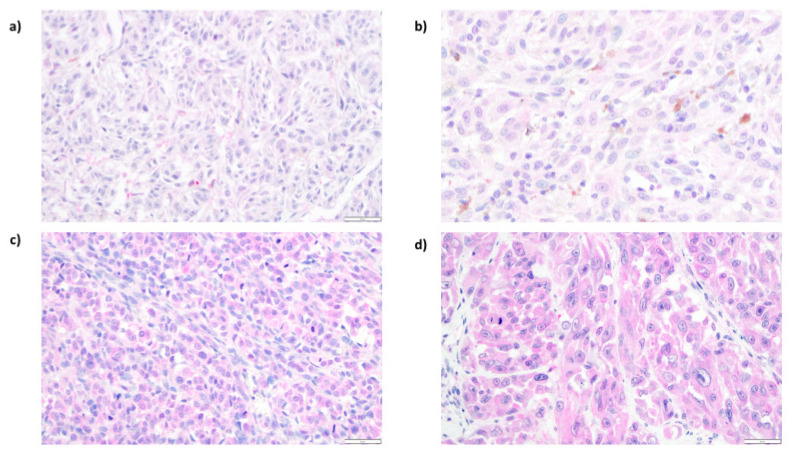
Representative results of immunohistochemical analysis of ENO1 expression in cutaneous melanoma patients. Low cytoplasmic ENO1 immunoreactivity in melanoma cells ((**a**), 200×; (**b**), 400×). High expression of ENO1 in tumoral cells ((**c**), 200×; (**d**), 400×).

ENO1 expression was evaluated by immunohistochemistry performed on tissue microarrays generated from 112 primary cutaneous melanomas (archival formalin-fixed, paraffin-embedded specimens). ENO1 immunoreactivity was measured with the H-score method. ENO1 H-scores ranged from 30 to 300, and the mean H-score value was 194 (±63.34), median: 202. In all positive cases, we observed predominantly cytoplasmic ENO1 subcellular distribution (Figure 4). For the statistical analysis, we divided the study group into two subgroups: (1) low ENO1 expression (defined as an H-score ≤200), and (2) high ENO1 expression (defined as an H-score >200). Low ENO1 immunoreactivity was observed in 56 patients (50%), and ENO1 overexpression was observed also in 56 patients (50%).

### 3.4. Analysis of Correlations between ENO1 Expression and Clinical Parameters 

**Figure 5:**
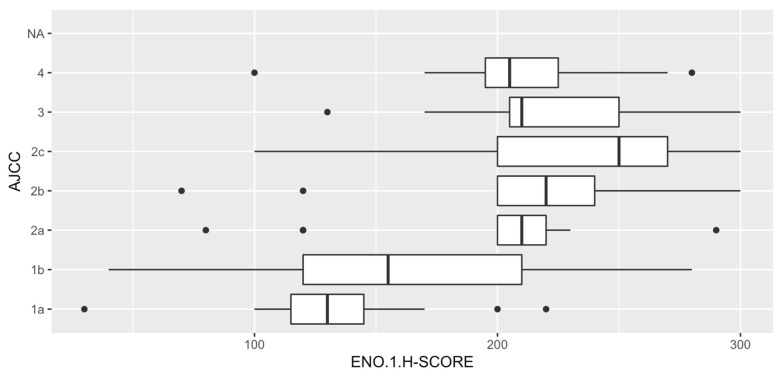
ENO1 expression and AJCC (American Joint Committee on Cancer) staging. The lowest ENO1 expression in tumoral cells was observed in patients with stage I cutaneous melanoma. In stages II-IV, it was observed a significant increasing of ENO1 immunoreactivity in neoplastic cells.

Overexpression of ENO1 in melanoma cells was significantly correlated with advanced stage of the disease—81% of patients with high expression of ENO1 were classified as pT3 or pT4 (*p* < 0.001). Low ENO1 immunoreactivity was strongly associated with lack of metastases in regional lymph nodes (*p* = 0.007) and lack of recurrence (*p* = 0.018) (Table 1). Moreover, 79% of patients (30/38) diagnosed at stage I according to the AJCC (8th edition) were characterized by low ENO1 immunoreactivity in melanoma cells (*p* < 0.001) (Figure 5).

### 3.5. Analysis of Correlations between ENO1 Expression and Histopathologic Parameters of Primary Tumors

Advanced primary tumors according to Breslow’s and Clark’s scales were characterized by overexpression of ENO1 (*p* < 0.001 for both scales). Furthermore, enhanced ENO1 immunoreactivity in melanoma cells was strongly correlated with high mitotic activity and presence of ulceration (*p* < 0.001, and *p* = 0.013, respectively). High tumoral immunologic response was predominantly observed in patients with reduced cytoplasmic ENO1 expression—64% of patients with brisk tumor-infiltrating lymphocytes were characterized by low ENO1 reactivity in melanoma cells (*p* = 0.039). Nodular melanomas, a histologic subtype of cutaneous melanoma with a worse outcome, revealed the highest level of ENO1 expression in comparison to superficial spreading and acral lentiginous melanomas (*p* < 0.001) (Table 2).

### 3.6. Impact of ENO1 Expression of Long-Term Prognosis of Cutaneous Melanoma Patients

**Figure 6:**
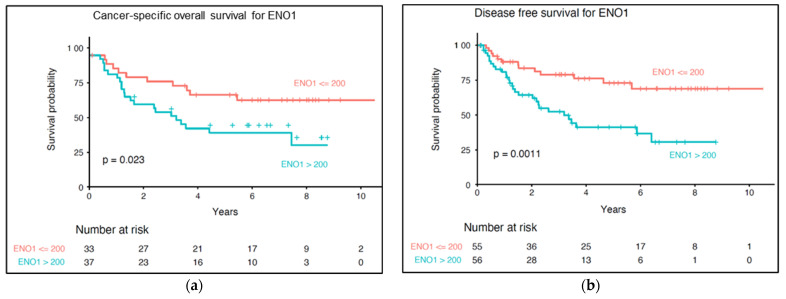
Kaplan–Meier analysis of the prognostic significance of ENO1 expression in cutaneous melanoma patients. Overexpression of ENO1 correlated with shorter cancer-specific overall survival (**a**) and shorter disease-free survival (**b**). *p* levels of the log-rank test.

Overexpression of ENO1 in tumor cells significantly correlated with shorter cancer-specific overall survival (*p* = 0.023) and disease-free survival (*p* = 0.001) (Figure 6). In univariate Cox regression model high ENO1 immunoreactivity had an important unfavorable impact on long-term survival (HR = 2.4, *p* = 0.027 for CSOS; and HR = 2.8, *p* = 0.002 for DFS) (Table 3).

**Figure 7:**
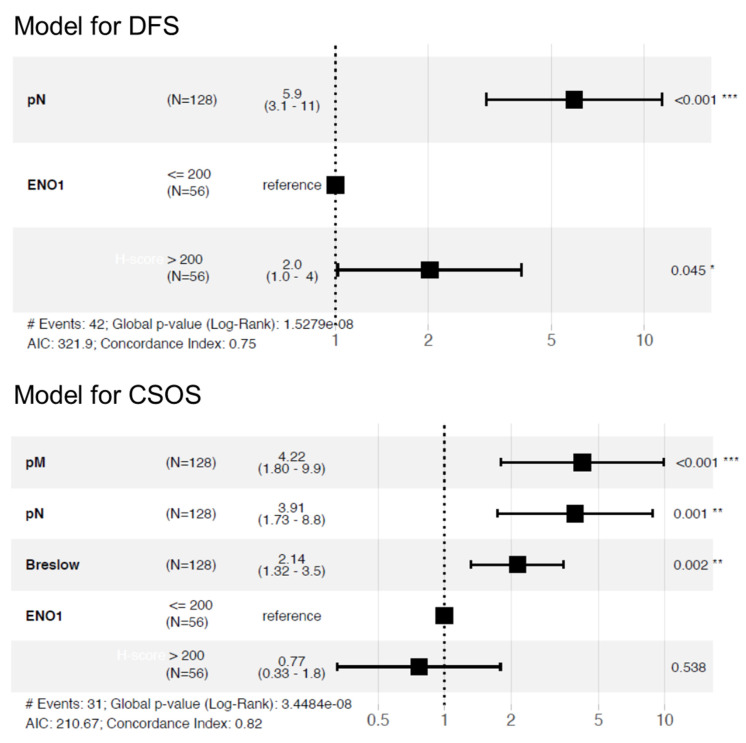
Multivariable regression model for disease-free survival and cancer-specific overall survival in cutaneous melanoma patients (DFS (disease-free survival), CSOS (cancer-specific overall survival).

The multivariable Cox regression model was created to test whether ENO1 expression may be used as an independent prognostic factor. After adjustment for regional lymph node status (HR: 5.9, 95% CI: 3.1−11.0, *p* < 0.001), high ENO1 expression was associated with shorter DFS (HR: 2.0, 95% CI: 1.0−4.0, *p* = 0.045) (Figure 7). When we comprehensively analyzed all the most clinically important parameters (Breslow thickness, nodal status, and distant metastases), ENO1 did not reach statistical significance (Figure 7).

## 4. Discussion

Our in vitro research revealed elevated expression of ENO1 protein and ENO1 enzymatic activity in four melanoma cell lines (A375, WM1341D, WM9, and Hs204T). Previously, overexpression of ENO1 transcript in the A375 cell line was described by Cecconi et al. [21]. In their study, downregulation of ENO1 achieved by the treatment of A375 cells with ascorbic acid led to reduction in cell fitness and migration capacities. Our current analysis of clinical melanoma samples in tissue microarrays also showed an increased expression of ENO1 in melanoma cells. Elevated expression of ENO1 in tumor cells in a cohort of 112 cutaneous melanoma patients correlated with unfavorable prognosticators such as high Breslow thickness, Clark level, increased mitotic activity, and presence of ulceration. Survival analysis revealed that overexpression of ENO1 was associated with shorter cancer-specific overall survival and shorter disease-free survival. 

Several glycolytic enzymes including ENO1 were found overexpressed in tumor cells subjected to hypoxia [25]. ENO1 has a crucial role in maintaining the Warburg effect, thus supporting cancer cell proliferation and formation of metastases [26,27]. Our research confirms previous authors’ findings about increased ENO1 activity in several solid cancers [19]. Interestingly, cell lines derived from the lymph node metastases (Hs294T, WM9) had significantly higher levels of ENO1 activity in hypoxia than in normoxia. This observation underlines an important role of ENO1 in tumor cells’ adaptation to cellular stress conditions. For example, when overexpressed in non-small cell lung cancer cell lines, ENO1 promoted cell glycolysis, growth, migration, and invasion [28]. Conversely, a knockdown of ENO1 in pancreatic, breast, and lung cancer cell lines induced an inhibition of cell cycle and the cell senescence [18]. 

To the best of our knowledge, this is the first study, which describes correlations between ENO1 expression and detailed clinical and pathologic parameters in cutaneous melanoma. The present analysis, performed on patients’ surgical resection specimens, showed that overexpression of ENO1 in tumoral cells was significantly correlated with disease advancement, the presence of metastases in regional lymph nodes, and shorter cancer-specific overall survival. Our clinical observations are in line with previously published clinical research on several other human cancers [29,30,31,32,33,34,35]. Proteomic analysis of peripheral T-cell lymphomas not otherwise specified (PTCL-NOS) revealed a significantly increased ENO1 level (eightfold) in neoplastic cells compared with the non-lymphoma tissue [29]. Moreover, PTCL-NOS patients with high expression of ENO1 had a worse prognosis [29]. In colorectal adenocarcinoma (CRC), Cheng et al. [31] showed that ENO1 overexpression was significantly correlated with the depth of tumor invasion, lymph node metastases, neural invasion, and TNM (Tumor-Node-Metastasis) staging, as well as with worse prognosis. Furthermore, knockdown of ENO1 significantly inhibited CRC cells proliferation and migration in in vitro analysis [31]. Functional analyses performed by Hu et al. [36] in CRC demonstrated that CD47 (a molecule which plays a crucial role in the immune escape of tumor cells, proliferation, and formation of metastases) directly interacted with ENO1 and protected it from ubiquitin-mediated degradation, subsequently promoting glycolytic activity and progression of CRC [36]. Similar prognostic results were observed in gastric cancer patients [32]. ENO1 overexpression in tumoral cells was significantly associated with nodal and distant metastases and increased level of ENO1 correlated with shorter overall survival. Interestingly, silencing of ENO1 suppressed Snail-induced epithelial-mesenchymal transition and inhibited the activation of transforming growth factor β (TGF-β) signaling pathway. Both pathways are crucial for progression of gastric cancer [32]. Moreover, ENO1 can be transferred between neoplastic cells via exosome-mediated crosstalk and exosome-derived ENO1 is essential to promote hepatocellular carcinoma growth, metastasis, and patient deterioration [35]. 

In our previous research, we examined the parameters of invasiveness of melanoma cell lines used in the current study [37,38,39,40]. We had shown that the A375 cell line exhibits the highest level of proliferation and cell migration (measured by relative wound density). This is in line with our clinical results, since melanoma cases with ENO1 overexpression were strongly correlated with high mitotic index (clinical equivalent of cell proliferation in vitro), and presence of nodal metastases (clinical equivalent of increased cellular migration). Cell lines derived from nodal metastases (WM9 and Hs294T) formed a higher number of adhesive structures supporting invasion called invadopodia, and were the most effective in degradation of extracellular matrix [37,38,39,40]. In the current study, we observed significantly higher levels of ENO1 activity in hypoxia than in normoxia in Hs294T and WM9. This result suggests that increased ENO1 activity under hypoxia, better reflecting lymph node milieu, may provide additional survival advantage to metastatic cells and help these cells to invade. Taken together our in vitro study demonstrated that A375, cell line with high biological aggressiveness, was characterized by the highest expression level and activity of ENO1. Moreover, hypoxia led to upregulation of ENO1 activity in two cell lines derived from lymph node metastases (WM9, Hs294T), but not in lines from primary skin lesions (A375, WM1341). This result suggests that increased ENO1 activity under hypoxia better reflecting lymph node milieu, and may provide additional survival advantage to metastatic cells.

There is an increasing number of studies reporting the overexpression of ENO1 in human cancers, making it a candidate for a promising therapeutic and diagnostic target in various types of cancers [9,41,42]. Zhang et al. [43] showed that using cinnamaldehyde (an active ingredient that originates from cinnamon) silences ENO1, arrests the cell cycle, and promotes apoptosis of melanoma cells [43]. The previously discussed ascorbic acid also interacts with ENO1 and induces the apoptosis of melanoma cells [21]. Interestingly, monoclonal antibody directed against ENO1 inhibited invasion, proliferation, and clone formation of cervical cancer cells, suggesting that ENO1mAb triggers promising anti-tumor effects [44]. In the future study, we will test the influence of alternatively spliced nuclear isoform of the ENO1–MBP−1 (a transcriptional repressor of multiple protooncogenes) on cutaneous melanoma cells proliferation and invasion [45].

## 5. Conclusions

In this research, the overexpression of ENO1 in the melanoma cell lines was correlated with the elevated invasiveness parameters of examined cells. Enhanced ENO1 expression in the cytoplasm of melanoma cells was correlated with unfavorable prognosticators such as Breslow thickness, Clark level, mitotic activity, presence of ulceration, and a worse prognosis in the analyzed cohort of patients. The variety of biological processes in which ENO1 plays an important function ensures areas for future studies. Our observations enable further ways for studies regarding a potential prognostic marker and therapeutic target in cutaneous melanoma.

## Figures and Tables

**Table 1:** Correlations between ENO1 expression and clinical parameters of cutaneous melanoma patients.

Clinical Parameters	ENO1 Expression		
	Low (H-Score ≤200) (N = 56)	High (H-Score >200) (N = 56)	*p* Value
**Age (18−86 years) ^a^**	64 (52–73)	65 (54–74)	0.40
**Gender ^b^**			1.00
Female	29 (52%)	28 (50%)	
Male	27 (48%)	28 (50%)	
**Primary tumor location ^c^**			0.063
Head/neck	2 (4%)	9 (16%)	
Extremities	22 (39%)	24 (43%)	
Trunk	31 (55%)	21 (38%)	
Hand/foot	1 (2%)	2 (4%)	
**Primary tumor (pT) ^a^**			**<0.001**
pT1	20 (36%)	5 (9%)	
pT2	13 (23%)	6 (11%)	
pT3	11 (20%)	16 (29%)	
pT4	12 (21%)	29 (52%)	
**Sentinel lymph node biopsy status (SNLB) ^b^**			**0.042**
No metastases (SNLB−)	19 (76%)	11 (44%)	
Metastases present (SNLB+)	6 (24%)	14 (56%)	
**Regional lymph nodes status (pN) ^b^**			**0.007**
Metastases absent (pN−)	49 (88%)	36 (64%)	
Metastases present (pN+)	7 (12%)	20 (36%)	
**Distant metastases (pM) ^b^**			0.53
No metastases (pM−)	52 (93%)	49 (88%)	
Metastases present (pM+)	4 (7%)	7 (12%)	
**AJCC (8th edition) stage ^a^**			**<0.001**
**I**	30 (54%)	8 (14%)	
**II**	17 (30%)	25 (45%)	
**III**	5 (9%)	16 (29%	
**IV**	4 (7%)	7 (12%)	
**Recurrence ^b^**			**0.018**
No	42 (75%)	29 (52%)	
Yes	14 (25%)	27 (48%)	

^a^*p* value of Wilcoxon two sample test; ^b^ *p* value of Fisher’s exact test; ^c^ *p* value of chi^2^ test.; statistically significant results (*p* < 0.05) are given in bold; American Joint Committee on Cancer (AJCC).

**Table 2:** Correlations between ENO1 expression and histopathological parameters in primary tumors of cutaneous melanoma patients.

Histopathological Parameters	ENO1 Expression		
	Low (H-Score ≤200) (N = 56)	High (H-Score >200) (N = 56)	*p* Value
**Breslow thickness ^a^**			**<0.001**
≤1 mm	20 (36%)	5 (9%)	
1.01−2.00 mm	13 (23%)	6 (11%)	
2.01−4.00 mm	11 (20%)	16 (29%)	
>4 mm	12 (21%)	29 (52%)	
**Clark level ^a^**			**<0.001**
I	0 (0%)	0 (0%)	
II	24 (43%)	5 (9%)	
III	17 (30%)	24 (43%)	
IV	11 (20%)	20 (36%)	
V	4 (7%)	7 (12%)	
**Histological type ^c^**			**<0.001**
Superficial spreading melanoma	35 (62%)	13 (23%)	
Nodular melanoma	20 (36%)	41 (73%)	
Acral lentiginous melanoma	1 (2%)	2 (4%)	
**Mitotic rate ^a^**			**<0.001**
0	22 (39%)	2 (4%)	
1−2	11 (20%)	5 (9%)	
>2	23 (41%)	49 (87%)	
**Ulceration ^c^**			**0.013**
No	39 (70%)	25 (45%)	
Yes	17 (30%)	21 (55%)	
**Lymphangioinvasion ^c^**			1.0
No	54 (96%)	52 (93%)	
Yes	2 (4%)	4 (7%)	
**Tumor-infiltrating lymphocytes ^c^**			**0.039**
No	5 (9%)	2 (4%)	
Non-brisk	28 (50%)	41 (73%)	
Brisk	23 (41%)	13 (23%)	
**Microsatellitosis ^c^**			1.00
No	54 (96%)	54 (96%)	
Yes	2 (4%)	2 (4%)	
**Regression ^c^**			1.00
No	53 (95%)	54 (96%)	
Yes	3 (5%)	2 (4%)	

^a^*p* of Wilcoxon two sample test; ^b^ *p* value of Fisher’s exact test; ^c^ *p* value of chi^2^ test; statistically significant results (*p* < 0.05) are given in bold.

**Table 3:** Univariate Cox proportional hazards model.

	N		Cancer-Specific Overall Survival			Disease-Free Survival	
		HR	95% CI	*p*-Value	HR	95% CI	*p*-Value
**Sex**	128	0.4	0.2−0.8	0.015 *	0.6	0.4−1.1	0.118
**Age**	128	3.3	1−10.4	0.043 *	2.0	0.8−4.7	0.126
**pN**	128	5.3	2.6−11	<0.001 *	7.3	4−13	<0.001 *
**pM**	128	3.5	1.6−7.4	<0.001 *	3.3	1.7−6.8	<0.001 *
**AJCC (8th edition) stage**	112	17.4	3.9−77.9	<0.001 *	12.5	4.7−33	<0.001 *
**Breslow thickness**	128	9.0	2.1−38.7	0.003 *	7.9	2.7−23	<0.001 *
**Clark level**	128	2.0	1.3−2.9	<0.001 *	1.8	1.4−2.5	<0.001 *
**Histologic type**	128	2.7	1.5−5	0.001 *	3.3	1.9−5.6	<0.001 *
**Ulceration**	128	2.9	1.5−6	0.003 *	2.3	1.3−4.1	0.005 *
**Lymphovascular invasion**	128	2.0	0.6−6.6	0.249	1.1	0.3−4.6	0.877
**TILs**	128	0.12	0.1−0.5	0.002 *	0.2	0.1−0.9	0.041 *
**Microsatellitosis**	128	3.1	1.1−8.9	0.035 *	3.5	1.3−9.9	0.016 *
**ENO1 H-score**	112	2.4	1.1−5	0.027 *	2.6	1.3−4.9	0.005 *

* *p* < 0.05, statistically significant; TILs (tumor-infiltrating lymphocytes).

## Data Availability

The data presented in this study are available on request from the corresponding author.

## Supplementary Materials

The following are available online at https://www.mdpi.com/article/10.3390/diagnostics12020254/s1, Figure S1. The expression level of ENO1 in the cellular extracts. Full immunoblots show ENO1 (48 kDa) and Akt 1/2/3 in the cell lysates on the same PVDF membrane. The cell lysates were prepared from melanoma cells lines derived from the primary skin lesions (A375, WM1341D) and lymph node metastases (Hs294T, WM9), and normal melanocytes (HEM). Figure S2. A standard curve of enolase activity measured spectrophotometrically at 570nm. Table S1. Full results of densitometric analysis of Western blotting for the ENO1 and Akt 1/2/3. The expression level was researched in four melanoma cell lines: A375, WM9, WM1341D, Hs294T, and one normal melanocyte: HEM. Table S2. Full results of enolase activity measured spectrophotometrically at 570nm in the melanoma cell lines: A375, Hs294T, WM1341D, and WM9, cultured in normoxic and hypoxic conditions.
