# Supplementary material for: Alpha-Enolase (ENO1) Correlates with Invasiveness of Cutaneous Melanoma—An In Vitro and a Clinical Study
Source: Diagnostics (Basel). 2022 Jan 20;12(2):254. doi: 10.3390/diagnostics12020254 (PMC8871300; doi:10.3390/diagnostics12020254)
Supplement: Supplementary file 1 [file diagnostics-12-00254-s001.zip › diagnostics-1500987-supplementary.pdf]

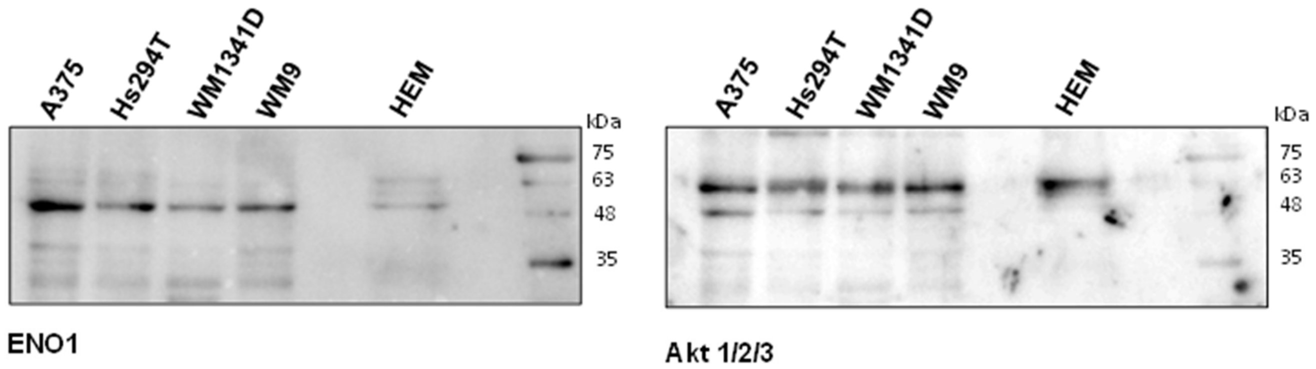

Supplementary Figure S1. The expression level of ENO1 in the cellular extracts. Full immunoblots show ENO1 (48 kDa) and Akt 1/2/3 in the cell lysates on the same PVDF membrane. The cell lysates were prepared from melanoma cells lines derived from the primary skin lesions (A375, WM1341D) and lymph node metastases (Hs294T, WM9), and normal melanocytes (HEM).

Supplementary Table S1. Full results of densitometric analysis of Western blotting for the ENO1 and Akt 1/2/3. The expression level was researched in four melanoma cell lines: A375, WM9, WM1341D, Hs294T, and normal melanocyte: HEM. (SEM (standard error of the mean))

**Cell line**	**Repetitions**	**ENO1**	**Akt 1/2/3**	**Protein levels**	**Mean protein levels**	**SEM**
**A375**	I	37.33	20.55	1.82	1.19	0.31
	II	5.54	6.36	0.87		
	III	5.66	6.37	0.89		
**Hs294T**	I	19.56	16.63	1.18	0.58	0.30
	II	2.29	7.64	0.30		
	III	2.13	7.71	0.28		
**WM1341D**	I	10.34	15.70	0.66	0.95	0.15
	II	7.47	6.69	1.12		
	III	7.06	6.57	1.08		
**WM9**	I	18.74	22.29	0.84	0.82	0.01
	II	7.12	8.83	0.81		
	III	7.23	8.92	0.81		
**HEM**	I	14.03	24.83	0.57	0.58	0.01
	II	4.85	8.16	0.59		

Supplementary Table S2. Full results of enolase activity measured spectrophotometrically at 570nm in the melanoma cell lines: A375, Hs294T, WM1341D, and WM9, cultured in normoxic and hypoxic conditions. (SEM (standard error of the mean))

Cell line	Repetitions	Normoxic			hypoxic			*p*-value
		Enolase activity [miliU/mg]	Mean [miliU/mg]	SEM	Enolase activity [miliU/mg]	Mean [miliU/mg]	SEM	
**A375**	I	525.80	595.28	26.40	472.39	583.77	39.43	ns
	II	636.36			639.68			
	III	582.58			583.33			
	IV	636.36			639.68			
**WM1341D**	I	426.53	492.77	25.33	401.50	500.91	35.15	ns
	II	532.40			550.63			
	III	479.76			500.87			
	IV	532.40			550.63			
**WM9**	I	341.14	394.38	20.34	378.97	469.51	31.96	0,047
	II	426.18			514.55			
	III	384.01			469.99			
	IV	426.18			514.55			
**Hs294T**	I	188.93	227.53	14.70	265.14	338.93	26.15	0,005
	II	250.45			376.09			
	III	220.27			338.41			
	IV	250.45			376.09			

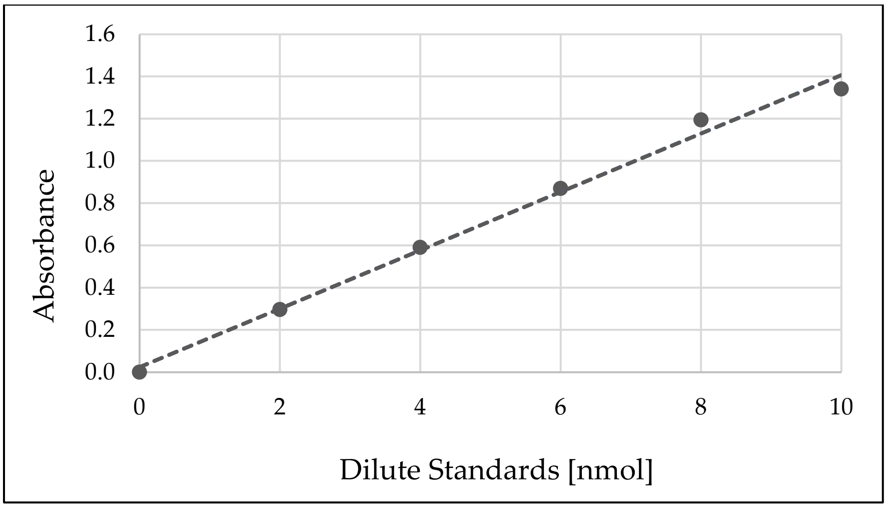

Supplementary Figure S2. A standard curve of enolase activity measured spectrophotometrically at 570nm.
